# 

**Published:** 1816-02

**Authors:** 


### MONTHLY CATALOGUE OF MEDICAL BOOKS.

A Treatise on the Mineral Waters of Gilsland. By W. Reid Clanny, M.D. M.R.I.A. &c. 8vo.—Callow.

Medical Transactions, published by the College of Physicians of London. Vol. V. 8vo.—Longman and Co.

Medico-Chirurgical Transactions, published by the Medical and Chirurgical Society of London. Volume the Sixth. 8vo.—Longman and Co.

Dialogues on Chemistry, intended for the Instruction and Entertainment of young People; in which the first Principles of that Science are fully explained. 2 vols. 18mo.—Baldwin and Co.

---

### *NOTICES TO CORRESPONDENTS.*

*We fear Dr. T.'s mode of announcing his work will have too much the appearance of an advertisement.*

*If the anonymous correspondent really did send such a specimen of Latinity as a prize composition, we recommend him to some grammar school in which the birch is not spared. If he intended it as a hoax, the Editors assure him they have a blanket in constant readiness, and their printer has always four stout pressmen, who may exalt such a witling higher than he wishes.*

*Communications have been received from* Drs. Ramsay, Kinglake, Sutton; E. C. B.; *and an anonymous paper on the Heart, &c.*

